# When to Devitalize

**Published:** 1906-06-15

**Authors:** J. E. Stoffer


### WHEN TO DEVITALIZE.

BY J. E. STOFFER, D.D.S.

Read before the Central Michigan Dental Society, February 23, 1906.

I didn't choose this subject because I thought I knew all about it, but because it was a subject about which but little has been said, and I fear too little thought. If I shall be able to set you thinking and draw out a good lively discussion, I shall feel that my efforts have not been in vain.

It is very easy to lay down a general rule as to devitalizing, and briefly stated it is this: "Devitalize whenever there is an exposed pulp." But there are so many exceptions to this rule that it requires considerable thought and experience to enable the operator to do the best for his patient.

A few months ago I saw in one of our Journals this statement "When in doubt devitalize," and I took exception to it for an error in that direction can never be rectified. Is it not better when in doubt to try to save the pulp? We have on the market several non-irritant non-conductive pulp capping material which, when wisely used, would save a large percent of pulps that are lost.

It is always best in those partially developed teeth that come to us with the pulp exposed to at least try to keep it alive if possible, for with the destruction of the pulp, all development of the tooth ceases, and it is impossible to fill the roots of these partially developed teeth with their large apical foramen so as to thoroughly class the opening and at the same time not to force the filling material through the end of the root to irritate and spoil the permanancy of the operation.

There are a large number of dentists who never hesitate to devitalize whenever the pulp is anywhere near encroached upon by decay, preferring to take the chances of thoroughly filling the canals, a thing which is impossible in the very fine or crooked canals. Of course there will be no immediate bad results, and the operator may flatter himself on the success of his operation, but wait a few years and he may not be so certain of the results. Then, too, there are those who devitalize teeth, and when the canals appear to be properly filled they never feel quite right, and are always just a little annoying, especially after catching cold. There are other operators who seldom devitalize if the exposure is too large, they remove the body of the pulp, cap the canals and insert their filling and it is surprising how many of such operations are successful, but I doubt if they are sufficiently numerous to warrant this procedure.

It seems to me that there is a medium between these two extremes that we might do well to follow. Unless there is at least an even chance for the pulp to remain healthy it is perhaps best to devitalize. We best serve our patients when we preserve the pulp of as many teeth as possible. It is best to err on the side of trying to save the pulp, for these errors can be remedied with but slight inconvenience to the patient, if he has been previously told that there is a possibility of the pulp dying and for him to return if the tooth should begin to trouble him.

I know there are those who claim that the pulp has served its purpose when once the tooth is fully developed, but this is hardly possible else nature would have provided some means of absorbing it. It seems to be one of nature's laws not to retain for any length of time anything that is useless. We have all observed that devitalized teeth are more brittle and much more susceptible to decay than live ones.

I fear there is a tendency especially among the unscrupulous to devitalize all teeth where there is a possible chance for the pulp to die. In the first place there is the added fee and in the second place if his work has been at all carefully done, he may be reasonably certain that his patient will not return within a few weeks or months with tooth sore or aching. But has he done the best possible for his patient? Certainly not. Of course, we all have patients where the above procedure is justifiably, for instance those impetuous unreasonable people who are always looking for a chance to find fault. In other words, the chronic kickers.

In all cases for crown or abutments for bridges, where the pulp is encroached upon by decay or excessive grinding so as to endanger its life, it is best to devitalize as it may save a considerable anoyance to the patient as well as the dentist. In most instances especially with teeth with more than one root, it is extremely difficult to open up and thoroughly fill the canals without removing the crown, a thing which is often impossible without the destruction of the crown. In case of good healthy pulp protected by considerable good healthy dentin, it is best to refrain from devitalizing. There seems to be a tendency for a devitalized tooth to elongate, possibly an effort on the part of the gums and alveolar process to throw off the dead tissue.

In conclusion, I would suggest that we think twice before sacrificing a pulp.

